# Do Reproductive Factors Influence T, N, and M Classes of Ductal and Lobular Breast Cancers? A Nation-Wide Follow-Up Study

**DOI:** 10.1371/journal.pone.0058867

**Published:** 2013-05-29

**Authors:** Seyed Mohsen Mousavi, Asta Försti, Kristina Sundquist, Kari Hemminki

**Affiliations:** 1 Division of Molecular Genetic Epidemiology, German Cancer Research Center (DKFZ), Heidelberg, Germany; 2 Center for Primary Health Care Research, Lund University/Region Skåne, Malmö, Sweden; Karolinska Institutet, Sweden

## Abstract

**Backgrounds:**

The clinical tumor-node-metastasis (T, N and M) classes of breast cancers provide important prognostic information. However, the possible association of TNM classes with reproductive factors has remained largely unexplored. Because every woman has a reproductive history, implications to outcome prediction are potentially significant.

**Methods:**

During the study period from 2002 through 2008, 5,614 pre- and 27,310 postmenopausal patients were identified in the Swedish Family-Cancer Database. Ordinal logistic regression analysis was used to estimate odds ratios (ORs) for TNM classes of breast cancers by histology. The reproductive variables were parity, age at first and last childbirth and time interval between first and last childbirth.

**Results:**

Among postmenopausal patients, the ORs for high-T class (T2–T4) (tumor size ≥2 cm) and metastasis were decreased by parity. A late age at first and last childbirth associated with high-T class and the effects were higher for lobular (OR for late age at first childbirth  = 2.85) than ductal carcinoma. Overall, long time interval between first and last childbirth was related to high-T class and metastasis. However, a short time interval between first and last childbirth in patients with late age at first or last childbirth increased the risk of metastasis. Late age at last childbirth was associated with increased occurrence of lobular carcinoma in situ. Among premenopausal ductal carcinoma patients, nulliparity and early age at first childbirth were associated with high-T class.

**Conclusions:**

Increasing parity was protective against high-T class and metastasis; late ages at first and last childbirth were risk factors for high-T class in postmenopausal breast cancers. The current decline in parity and delayed age at first childbirth in many countries may negatively influence prognosis of breast cancer.

## Introduction

Demographic changes over recent decades in developed countries and even among educated women in developing countries have modified reproductive patterns towards delayed childbearing and declining parity [1]–[4]. Reproductive factors are strongly associated with the risk of breast cancer and, unfortunately, both delayed childbearing and low parity increase the risk of breast cancer [5]. In fact, in developed countries these two reproductive parameters may explain as much as 30 to 40% of breast cancer etiology [6]. Many studies have also shown that reproductive factors differentially influence breast cancer histology [7], [8]. For example, multiparity is known to decrease the risk of ductal and lobular carcinomas, while late age at first childbirth increases the risk [7]–[11]. The influence of age at last childbirth and birth interval remains unclear [12]–[14]. The underlying mechanisms are not well known but, it is widely believed that hormonal and other physiological changes in the mammary gland and rapid proliferation of breast tissue during pregnancy and lactation play a major role [9], [15]–[17].

In developed countries, breast cancer is mainly a postmenopausal disease. The effects of reproductive factors on histology of breast cancer vary according to the menopausal status [8], [18]. Among postmenopausal women, the effects of reproductive factors are more strongly associated with invasive ductal than lobular carcinoma [19]. However, for in situ breast cancer the reverse may be true [20], [21]. Among premenopausal women, reproductive risk factors may be more strongly associated with in situ than invasive cancers [18], [22].

Only a few previous studies have examined the effect of reproductive factors on the stage or grade of histology-specific breast cancer [12], [20], [21]. These suggest that nulliparity, late age at first childbirth, and the first two years after pregnancy are associated with unfavorable clinical data. To our knowledge, no previous study has analyzed the association of reproductive factors with tumor-node-metastasis (T, N and M) classes of breast cancer which provide important prognostic information [23]–[25]. We explore here the association of parity, age at first and last childbirth, and time interval between first and last childbirth with the TNM classes of ductal and lobular carcinomas, the most common histological subtypes [5]. Both pre- and postmenopausal breast cancers were included from the Swedish Cancer Registry from 2002 through 2008.

## Patients and Methods

We used the 2010 update of the Swedish Family-Cancer Database (FCD), which contains population-based data from the Swedish Cancer Registry, multigenerational registries, national censuses and death notifications [26], [27]. This database contains information on people born in Sweden since 1932 and their biological parents. Native Swedish women were defined as those who, along with their parents, were born in Sweden.

Following the start of cancer registration in 1958, data on cancers in the FCD had a 4-digit diagnostic code according to the seventh revision of the International Classification of Disease (ICD). The codes for breast cancer were 170.1 and 170.2. Since 1993, ICD-O-2/ICD with histopathological data according to the Systematized Nomenclature of Medicine (SNOMED) has been used. This coding system gives a detailed tumor histology-topology. We analyzed ductal (SNOMED = 8500 and 8501), lobular (8520), tubular (8211) and mucinous (8480) carcinomas. Nonspecific adenocarcinoma (8140) was not included in our analysis (N = 2,493). All breast cancer cases (100%) registered in the Cancer Registry were histologically verified [28].

Tumor size and local growth (T), regional lymph node involvement (N) and the presence of metastasis (M) according to the TNM classification system published by the American Joint Committee on Cancer have been available in the FCD since 2002 [6], [29]. The basis for TNM classification in the FCD was clinical or pathological findings [28]. Numbers or letters after T, N and M provide more details about the classification. The numbers 0 through 4 indicate increasing severity. Breast cancer patients in native Swedish women from 2002 to 2008 were obtained from the FCD.

Parity (0, 1, 2, 3 and ≥4), age at first and last childbirth (≤19, 20–29, 30–39 and ≥40), and time interval between first and last childbirth (1–4, 5–9 and ≥10) were obtained from the FCD. The data for ages at menarche and menopause were not available in the dataset. It also contains longitudinal demographic and socio-economic data from the national censuses of 1960, 1970, 1980, and 1990 [30]. The occupational data were grouped into white-collar workers, blue-collar workers, privates, professionals and others. We also divided Sweden regionally into three groups: North, South and large cities.

Ordinal logistic regression was used to calculate odds ratios (ORs) (PROC LOGISTIC; SAS software version 9.2; SAS Institute Inc., Cary, NC, USA). The parity (number of children = 1), the age at first and last childbirth (20–29 years), and the time interval between first and last childbirth (1–4 years) were selected as references. The ORs were adjusted for age at diagnosis, region, occupation and, in some analyses, parity. To assess the effect of the median age at menopause, the analyses were stratified by age (≤50 and >50 years) [31], [32]. Confidence intervals (95% CI) were calculated assuming a Poisson distribution using T1, N0 and M0 as the references for “Tis” (carcinoma in situ) and “T2–T4”, “N1–N3” and “M1”, respectively. There was missing information on 7,557 patients for T, on 8,030 patients for N and on 8,124 patients on M in our database. We also excluded TX (N = 1,033), T0 (1,673), NX (1,753) and MX (5,785) from our analysis. A P-value of less than 0.05 was considered statistically significant.

Our study population comprised 23,045 ductal, 4,611 lobular, 1,047 tubular and 728 mucinous carcinoma patients. The analyses of the last two histologies are not presented in this report because of the small numbers of cases in the TNM classes for menopausal status and any reproductive factors. As there are correlations between the variables age at first and last childbirth and time interval between first and last childbirth, we stratified age at first and last childbirth by time interval between first and last childbirth. Alternatively, we examined the effect of parity and the time interval between first and last childbirth by adding the age at first or last childbirth to the adjustment; however, because of the small numbers of premenopausal breast cancer cases in any subclasses, this analysis was only applied to postmenopausal cases.

### Ethics Statement

The Lund regional Ethics Committee approved the study protocol. The corresponding author had full access to the FCD and had final responsibility for the decision to submit for publication.

## Results

We identified 27,310 postmenopausal and 5,614 premenopausal breast cancer patients in the database. The histological type of breast cancer among postmenopausal patients was ductal in 68.5% of patients and lobular in 14.8% of patients, while premenopausal carcinomas were 77.1% ductal and 10.1% lobular.

Our data included 10,794 T1, 13,767 N0 and 15,186 M0 cases of postmenopausal breast cancer (Table 1). Parity had no significant influence on carcinoma in situ. For invasive cancer, we calculated ORs for any T class separately. The results were very similar for any of them. Therefore, to catch the maximum number of cases for each group, we combined T2–T4. The OR decreased for higher T classes (T2–T4) among patients with two (0.82) or three (0.83) children compared to patients with one child. Multiparity was associated with a decreased risk of distant metastases (ORs ranging from 0.65 to 0.70). For invasive breast cancer, increasing age at first and last childbirth above 30 years were associated with a higher T class; the OR was 1.84 and 1.45 for those with an age at first and last childbirth over 39 years, respectively. Similarly, a time interval between first and last childbirth ≥10 years associated with an increasing T. Patients with an age at last childbirth ≥40 years were at an increased risk of lymph node involvement (1.22), while those with a time interval between first and last childbirth ≥5 years had a risk of distant metastases (1.40 for time interval between first and last childbirth 5–9 years). Risk of metastasis was also increased for cases with an age at first childbirth ≤19 years (1.34).

**Table 1:** Odds ratios (ORs) for TNM classes in postmenopausal breast cancer by reproductive factors.

Reproductive factors	T1	Tis* vr. T1			T2–T4** vr. T1			N0	N1–N3 vr. N0			M0	M1 vr. M0		
	N	N	OR (95%CI)	P	N	OR (95%CI)	P	N	N	OR (95%CI)	P	N	N	OR (95%CI)	P
*Parity^1^*															
1 (Ref.)	1,924	39	1.00		1,620	1.00		2,546	1,005	1.00		2,862	145	1.00	
0	1,114	26	1.16 (0.70–1.93)	0.57	816	1.08 (0.96–1.21)	0.21	1,527	552	0.97 (0.85–1.09)	0.58	1,638	55	0.90 (0.65–1.24)	0.51
2	4,806	107	1.16 (0.80–1.68)	0.44	3,043	**0.82** (**0.76–0.89**)	<0.0001	6,030	2,160	0.91 (0.84–1.00)	0.05	6,572	199	**0.65** (**0.52–0.82**)	<0.01
3	2,185	50	1.19 (0.78–1.82)	0.43	1,457	**0.83** (**0.75–0.91**)	<0.01	2,676	1,070	0.99 (0.90–1.10)	0.87	2,943	100	**0.69** (**0.53–0.89**)	0.01
≥4	765	12	0.79 (0.41–1.52)	0.47	687	0.98 (0.86–1.11)	0.73	988	435	1.03 (0.90-1.18)	0.68	1,171	49	**0.70** (**0.50–0.98**)	0.04
Total	10,794	234			7,623			13,767	5,222			15,186	548		

*Age at first childbirth* (*year*)*^2^*															
20–29 (Ref.)	6,932	148	1.00		4,714	1.00		8,651	3,277	1.00		9,504	322	1.00	
≤19	1,208	30	1.16 (0.77–1.73)	0.48	742	0.93 (0.84–1.03)	0.18	1,485	601	1.05 (0.95–1.16)	0.37	1,658	74	**1.34** (**1.03–1.73**)	0.03
30–39	1,468	30	0.89 (0.59–1.32)	0.56	1,260	**1.20** (**1.10–1.31**)	<0.0001	1,998	740	0.99 (0.90–1.09)	0.79	2,252	92	1.20 (0.94–1.52)	0.14
≥40	72	0	(−)		91	**1.84** (**1.33–2.53**)	<0.01	106	52	1.35 (0.96–1.88)	0.08	134	5	1.14 (0.46–2.82)	0.78
Total	9,680	208			6,807			12,240	4,670			13,548	493		

*Age at last childbirth* (*year*)*^2^*															
20–29 (Ref.)	4,503	101	1.00		2,822	1.00		5,576	2,079	1.00		6,101	207	1.00	
≤19	163	4	1.07 (0.39–2.95)	0.90	119	1.12 (0.87–1.43)	0.38	223	79	0.95 (0.73–1.24)	0.70	250	11	1.25 (0.67–2.33)	0.48
30–39	4,626	95	0.88 (0.66–1.17)	0.38	3,455	**1.11** (**1.04–1.18**)	<0.01	5,927	2,272	1.02 (0.95–1.10)	0.56	6,544	246	1.06 (0.88–1.28)	0.55
≥40	388	8	0.83 (0.40–1.74)	0.63	411	**1.45** (**1.25–1.69**)	<0.0001	514	240	**1.22** (**1.03–1.43**)	0.02	653	29	1.12 (0.75–1.68)	0.58
Total	9,680	208			6,807			12,240	4,670			13,548	493		

*Time interval between first and last childbirth* (*year*)*^2^*															
1–4 (Ref.)	3,429	73	1.00		2,068	1.00		4,259	1,536	1.00		4,640	112	1.00	
5–9	2,762	64	1.12 (0.80–1.58)	0.50	1,833	1.03 (0.95–1.12)	0.52	3,397	1,318	1.05 (0.96–1.14)	0.32	3,714	141	**1.40** (**1.08–1.80**)	0.01
≥10	1,524	31	0.91 (0.59–1.40)	0.66	1,268	**1.20** (**1.09–1.32**)	<0.01	1,987	798	1.05 (0.95–1.16)	0.35	2,279	94	**1.39** (**1.05–1.84**)	0.02
Total	7,715	168			5,169			9,643	3,652			10,633	347		

Bold type: 95% CI does not include 1.00. The ORs were adjusted for:

1. Age at diagnosis, region, and occupation.

2. Age at diagnosis, region, occupation, and parity.

* Carcinoma in situ.

** The number of cases for T2 (N = 6,098), T3 (871), and T4 (654).

The analyses shown in Table 1 were repeated in Table 2 by adjusting for parity and by stratifying for time interval between first and last childbirth. Risk of distant metastases was increased in patients with late age at first and last childbirth when children were born in a short interval of 1–4 years; the highest significant OR was 1.85 for an age at last childbirth of 30–39 years. A high-T class was observed for patients with a late age at first and last childbirth when the time interval between first and last childbirth was 5–9 years. The OR for high-T class was 0.77 for patients with early age at first childbirth and a time interval between first and last childbirth of more than 9 years. Additional analyses of parity and time interval between first and last childbirth showed that adjustment for age at first or last childbirth did not change the results (data not shown).

**Table 2:** Odds ratios (ORs) for TNM classes in postmenopausal breast cancer by age at first and last childbirth, stratified by time interval between first and last childbirth.

Age at breast cancer diagnosis	T1	Tis* vr. T1			T2–T4** vr. T1			N0	N1–N3 vr. N0			M0	M1 vr. M0		
	N	N	OR (95%CI)	P	N	OR (95%CI)	P	N	N	OR (95%CI)	P	N	N	OR (95%CI)	P
*Time interval between first and last childbirth 1-4 yearsAge at first childbirth (year)*															
20-29 (Ref.)	2,574	55	1.00		1,503	1.00		2,607	1,145	1.00		2,863	75	1.00	
≤19	277	5	0.84 (0.33–2.13)	0.71	138	0.08 (0.71–1.10)	0.25	20	132	1.14 (0.92–1.42)	0.24	23	5	0.63 (0.25–1.58)	0.33
30–39	569	13	1.00 (0.54–1.87)	1.00	415	1.14 (0.99-1.32)	0.09	1,574	252	0.90 (0.77–1.06)	0.21	1,679	31	**1.61 (1.04–2.50)**	0.03
≥40	9	0			12	2.22 (0.91–5.40)	0.08	58	7	1.79 (0.70–4.58)	0.23	75	1	2.96 (0.37–23.62)	0.31
Total	3,429	73			2,068			1,574	1,536			4,640	112		

Age at last childbirth (year)															
20–29 (Ref.)	2,148	49	1.00		1,212	1.00		2,607	948	1.00		2,863	53	1.00	
≤19	19	0			8	0.69 (0.29–1.60)	0.37	20	7	0.92 (0.39–2.20)	0.86	23	0		
30–39	1,219	23	0.81 (0.48–1.35)	0.42	809	1.09 (0.97–1.22)	0.18	1,574	556	0.99 (0.87–1.12)	0.84	1,679	56	1.85 (1.25–2.74)	<0.01
≥40	43	1	0.81 (0.11–6.22)	0.84	39	1.40 (0.89–2.19)	0.16	58	25	1.19 (0.73–1.92)	0.49	75	3	1.96 (0.58–6.57)	0.28
Total	3,429	73			2,068			4,259	1,536			4,640	112		

Time interval between first and last childbirth 5–9 years															

Age at first childbirth (year)															
20–29 (Ref.)	2,142	51	1.00		1,368	1.00		2,595	1,005	1.00		2,826	104	1.00	
≤19	370	10	1.17 (0.58–2.37)	0.66	219	1.00 (0.83–1.20)	0.99	451	188	1.08 (0.89–1.30)	0.44	482	25	1.60 (1.01–2.53)	0.05
30–39	249	3	0.46 (0.14–1.49)	0.20	245	1.48 (1.22–1.80)	<.0001	350	124	0.92 (0.74–1.15)	0.48	404	12	0.75 (0.41–1.39)	0.36
≥40	1	0			1	1.61 (0.1–27.32)	0.74	1	1	2.49 (0.16–40.04)	0.52	2	0		
Total	2,762	64			1,833			3,397	1,318			3,714	141		

Age at last childbirth (year)															
20–29 (Ref.)	1,130	28			667	1.00		1,387	534	1.00		1,483	66	1.00	
≤19	0	0	1.00		0			0	0			0	0		
30–39	1,577	35	0.84 (0.50–1.41)	0.51	1,104	1.14 (1.01–1.30)	0.04	1,937	749	1.02 (0.89–1.17)	0.78	2,142	70	0.73 (0.51–1.03)	0.08
≥40	55	1	0.66 (0.09–5.01)	0.68	62	1.73 (1.18–2.55)	0.01	73	35	1.28 (0.84–1.96)	0.25	89	5	1.22 (0.47–3.16)	0.68
Total	2,762	64			1,833			3,397	1,318			3,714	141		

Time interval between first and last childbirth ≥10 years															
Age at first childbirth (year)															
20–29 (Ref.)	1,077	18	1.00		953	1.00		1,416	571	1.00		1,625	60	1.00	
≤19	417	11	1.46 (0.67–3.20)	0.34	274	0.77 (0.64–0.93)	0.01	525	209	0.99 (0.82–1.20)	0.90	598	33	1.56 (1.00–2.44)	0.05
30–39	30	2	3.78 (0.80–17.9)	0.09	41	1.34 (0.82–2.18)	0.25	46	18	0.97 (0.55–1.69)	0.90	56	1	0.41 (0.06–3.07)	0.39
≥40	0	0			0			0	0			0	0		
Total	1,524	31			1,268			1,987	798			2,279	94		

Age at last childbirth (year)															
20–29 (Ref.)	1,077	0	1.00		53	1.00	112	41	1.00		131	5	1.00		0.96
≤19	417	0			0		0	0			0	0		0.96
30–39	30	25			983	1.22 (0.85–1.75)	0.28	1,585	621	1.07 (0.74–1.55)	0.73	1,775	72	0.98 (0.38–2.48)	0.96
≥40	0	6			232	1.41 (0.95–2.11)	0.09	290	136	1.28 (0.84–1.94)	0.25	373	17	1.00 (0.35–2.80)	0.99
Total	1,524	31		1,268			1,987	798			2,279	94			

Bold type: 95% CI does not include 1.00.

Women with two or more children were included. ORs were adjusted for parity, region and occupation.

* Carcinoma in situ.

Data for postmenopausal ductal cancer are shown in Table 3. As nearly 70% of all breast cancers were ductal, the ORs were almost identical to those shown in Table 1. In contrast, many of the significant effects shown in Table 1 were reinforced for lobular cancer (Table 4). These included decreased ORs for high-T class (0.77 and 0.72 when parity was 2 or 3, respectively, reversely, when parity was equal or greater than 4, the OR for T4 class increased: N = 13, OR = 2.75, 95% CI: 1.12–6.73), increased ORs for high-T class according to age at first and last childbirth (2.85 when age at first childbirth was over 39 years) and an increased OR for distant metastases (2.23 when age at first childbirth was less than 20 years). A novel effect was noted for lobular carcinoma in situ for which the OR was 7.86 when age at last childbirth was 30–39 years.

**Table 3:** Odds ratios (ORs) for TNM classes in postmenopausal ductal carcinoma by reproductive factors.

Reproductive factors	T1	Tis* vr. T1			T2–T4** vr. T1			N0	N1–N3 vr. N0			M0	M1 vr. M0		
	N	N	OR (95%CI)	P	N	OR (95%CI)	P	N	N	OR (95%CI)	P	N	N	OR (95%CI)	P
*Parity^1^*															
1 (Ref.)	1,399	22	1.00		1,033	1.00		1,746	717	1.00		1,979	72	1.00	
0	830	20	1.54 (0.83–2.87)	0.17	553	1.08 (0.95–1.25)	0.25	1,115	402	0.92 (0.80–1.07)	0.29	1,211	30	0.90 (0.58–1.41)	0.65
2	3,519	71	1.32 (0.82–2.15)	0.26	2,004	**0.84** (**0.76–0.93**)	<0.01	4,247	1606	0.93 (0.84–1.03)	0.17	4,699	105	**0.67** (**0.49–0.91**)	0.01
3	1,599	31	1.31 (0.75–2.28)	0.34	958	**0.85** (**0.75–0.95**)	<0.01	1,871	784	1.00 (0.89–1.13)	0.95	2,083	55	0.74 (0.52–1.06)	0.10
≥4	534	7	0.92 (0.39–2.18)	0.85	440	1.03 (0.88–1.20)	0.73	669	307	1.03 (0.88–1.21)	0.73	799	30	0.84 (0.54–1.30)	0.44
Total	7,881	151			4,988			9,648	3,816			10,771	292		

*Age at first childbirth* (*year*)*^2^*															
20–29 (Ref.)	5,029	98	1.00		3,094	1.00		6,027	2394	1.00		6,724	176	1.00	
≤19	904	20	1.09 (0.67–1.78)	0.73	517	0.95 (0.84–1.07)	0.41	1,077	471	1.08 (0.96–1.22)	0.19	1,227	39	1.20 (0.84–1.71)	0.32
30–39	1,064	13	0.59 (0.33–1.06)	0.08	772	**1.13** (**1.02–1.26**)	0.02	1,359	512	0.96 (0.85–1.07)	0.42	1,526	44	1.13 (0.81–1.59)	0.48
≥40	54	0	(−)		52	**1.51** (**1.02–2.24**)	0.04	70	37	1.37 (0.91–2.05)	0.13	83	3	1.46 (0.45–4.70)	0.53
Total	7,051	131			4,435			8,533	3,414			9,560	262		

*Age at last childbirth* (*year*)*^2^*															
20–29 (Ref.)	3,327	72	1.00		1,891	1.00		3,936	1562	1.00		4,401	111	1.00	
≤19	125	2	0.72 (0.17–3.00)	0.65	85	1.16 (0.87–1.55)	0.31	168	64	0.96 (0.72–1.29)	0.80	194	4	0.79 (0.29–2.17)	0.65
30–39	3,328	52	0.73 (0.51–1.06)	0.10	2,211	**1.10** (**1.01–1.19**)	0.02	4,080	1628	1.00 (0.92–1.08)	0.95	4,544	132	1.13 (0.87–1.47)	0.34
≥40	271	5	0.82 (0.33–2.06)	0.67	248	**1.40** (**1.16–1.68**)	<0.01	349	160	1.12 (0.92–1.37)	0.25	421	15	1.26 (0.72–2.19)	0.42
Total	7,051	131			4,435			8,533	3,414			9,560	262		

Bold type: 95% CI does not include 1.00. The ORs were adjusted for:

1. Age at diagnosis, region, and occupation.

2. Age at diagnosis, region, occupation, and parity.

* Carcinoma in situ.

** The number of cases for T2 (N = 4,133), T3 (484), and T4 (371).

**Table 4:** Odds ratios (ORs) for TNM classes in postmenopausal lobular carcinoma by reproductive factors.

Reproductive factors	T1	Tis* vr. T1			T2–T4** vr. T1			N0	N1–N3 vr. N0			M0	M1 vr. M0		
	N	N	OR (95%CI)	P	N	OR (95%CI)	P	N	N	OR (95%CI)	P	N	N	OR (95%CI)	P
*Parity^1^*															
1 (Ref.)	246	3	1.00		303	1.00		404	164	1.00		459	19	1.00	
0	146	2	1.04 (0.17–6.49)	0.96	152	0.95 (0.72–1.27)	0.75	216	82	0.98 (0.71–1.35)	0.91	229	6	0.74 (0.29–1.92)	0.54
2	618	11	1.61 (0.44–5.93)	0.47	555	**0.77** (**0.62–0.94**)	0.01	858	320	0.90 (0.72–1.13)	0.37	950	28	0.75 (0.41–1.36)	0.34
3	318	6	1.57 (0.38–6.45)	0.53	277	**0.72** (**0.57–0.91**)	0.01	430	178	0.94 (0.73–1.22)	0.66	471	19	1.01 (0.52–1.94)	0.98
≥4	92	4	2.83 (0.6–13.32)	0.19	130	1.04 (0.75–1.44)	0.80	138	78	1.20 (0.85–1.68)	0.30	181	6	0.72 (0.28–1.85)	0.49
Total	1,420	26			1,417			2,046	822			2,290	78		

*Age at first childbirth* (*year*)*^2^*															
20–29 (Ref.)	919	17	1.00		846	1.00		1,293	513	1.00		1,423	40	1.00	
≤19	152	2	0.65 (0.14–2.88)	0.56	125	0.89 (0.69–1.16)	0.39	203	79	0.94 (0.71–1.26)	0.69	226	15	**2.23** (**1.20–4.16**)	0.01
30–39	194	5	1.29 (0.46–3.64)	0.63	271	**1.43** (**1.16–1.77**)	<0.01	315	137	1.11 (0.88–1.40)	0.36	383	17	1.50 (0.83–2.71)	0.17
≥40	9	0			23	**2.85** (**1.29–6.28**)	0.01	19	11	1.40 (0.65–3.00)	0.39	29	0		
Total	1,274	24			1,265			1,830	740			2,061	72		

*Age at last childbirth* (*year*)*^2^*															
20–29 (Ref.)	565	2	1.00		494	1.00		801	301	1.00		876	32	1.00	
≤19	19	0			15	0.85 (0.42–1.72)	0.66	29	8	0.75 (0.33–1.66)	0.47	31	1	0.82 (0.11–6.32)	0.85
30–39	628	20	**7.86** (**1.81–34.20**)	0.01	662	1.14 (0.96–1.34)	0.14	904	381	1.10 (0.91–1.32)	0.32	1,020	34	0.88 (0.53–1.45)	0.61
≥40	62	2	**8.08** (**1.07–61.15**)	0.04	94	**1.51** (**1.06–2.15**)	0.02	96	50	1.23 (0.85–1.80)	0.27	134	5	0.85 (0.32–2.28)	0.75
Total	1,274	24			1,265			1,830	740			2,061	72		

*Time interval between first and last childbirth* (*year*)*^2^*															
1–4 (Ref.)	439	4	1.00		389	1.00		609	233	1.00		679	16	1.00	
5–9	369	9	2.59 (0.78–8.66)	0.12	338	0.98 (0.80–1.20)	0.83	501	209	1.03 (0.82–1.29)	0.81	560	24	1.77 (0.93–3.40)	0.08
≥10	214	7	2.94 (0.83–10.44)	0.10	232	1.10 (0.86–1.39)	0.45	308	132	0.99 (0.76–1.28)	0.91	355	12	1.22 (0.56–2.64)	0.62
Total	1,022	20			959			1,418	574			1,594	52		

Bold type: 95% CI does not include 1.00. The ORs were adjusted for:

1. Age at diagnosis, region, and occupation.

2. Age at diagnosis, region, occupation, and parity.

* Carcinoma in situ.

** The number of cases for T2 (N = 1,106), T3 (236), and T4 (75).

The database included 4,328 cases of premenopausal ductal carcinoma, accounting for 77% of premenopausal cancers (Table 5). The main differences to postmenopausal ductal carcinoma were that for premenopausal cancer nulliparity associated with increased risk of high-T class (1.44). It was mainly caused by T4 class (N = 31, OR = 3.37, 95%CI: 1.66–6.85). High-T class was also increased in patients with age at first childbirth of less than 20 years whereas age at first childbirth of over 39 years was not a risk factor. Within the limits of detection, no reproductive parameter changed the ORs for carcinoma in situ or for lymph node or distant metastases.

**Table 5:** Odds ratios (ORs) for TNM classes in premenopausal ductal carcinoma by reproductive factors.

Reproductive factors	T1	Tis* vr. T1			T2–T4** vr. T1			N0	N1–N3 vr. N0			M0	M1 vr. M0			
	N	N	OR (95%CI)	P	N	OR (95%CI)	P	N	N	OR (95%CI)	P	N	N	OR (95%CI)	P	
*Parity^1^*																
1 (Ref.)	280	5	1.00		207	1.00		330	174	1.00		392	8	1.00		
0	235	8	1.79 (0.57–5.59)	0.32	252	**1.44** (**1.11–1.85**)	–0.01	343	180	0.99 (0.76–1.28)	0.93	408	14	1.67 (0.69–4.04)	0.26	
2	830	24	1.67 (0.63–4.45)	0.30	528	0.86 (0.70–1.06)	0.16	949	481	0.97 (0.78–1.20)	0.76	1,079	33	1.46 (0.67–3.19)	0.35	
3	358	8	1.39 (0.45–4.36)	0.57	245	0.94 (0.73–1.20)	0.59	393	235	1.14 (0.89–1.46)	0.30	488	8	0.77 (0.28–2.09)	0.61	
≥4	100	2	1.33 (0.25–7.11)	0.74	56	0.77 (0.53–1.13)	0.18	108	58	1.04 (0.72–1.52)	0.83	131	2	0.75 (0.15–3.62)	0.72	
Total	1,803	47			1,288			2,123	1,128			2,498	65			

*Age at first childbirth* (*year*)*^2^*															
20–29 (Ref.)	1,037	30	1.00		640	1.00		1,159	602	1.00		1,355	37	1.00	
≤19	79	2	0.98 (0.22–4.28)	0.97	66	**1.43** (**1.01–2.03**)	0.04	94	56	1.18 (0.83–1.68)	0.35	114	2	0.64 (0.15–2.75)	0.55
30–39	438	6	0.48 (0.20–1.19)	0.12	324	**1.25** (**1.04–1.50**)	0.02	511	283	1.10 (0.92–1.32)	0.30	606	12	0.81 (0.41–1.59)	0.54
≥40	14	1	2.55 (0.31–21.04)	0.38	6	0.80 (0.30–2.10)	0.65	16	7	0.90 (0.37–2.23)	0.83	15	0		
Total	1,568	39			1,036			1,780	948			2,090	51		

*Age at last childbirth* (*year*)*^2^*															
20–29 (Ref.)	538	14	1.00		345	1.00		605	314	1.00		706	24	1.00	
≤19	7	0			8	1.87 (0.66–5.29)	0.24	13	4	0.67 (0.21–2.08)	0.48	15	0		
30–39	973	23	1.07 (0.54–2.12)	0.86	640	1.10 (0.92–1.30)	0.30	1,094	598	1.10 (0.93–1.31)	0.28	1,288	27	0.69 (0.39–1.21)	0.19
≥40	50	2	1.85 (0.40–8.59)	0.43	43	**1.58** (**1.02–2.44**)	0.04	68	32	1.04 (0.66–1.62)	0.88	81	0		
Total	1,568	39			1,036			1,780	948			2,090	51		

*Time interval between first and last childbirth* (*year*)*^2^*															
1–4 (Ref.)	711	18	1.00		463	1.00		814	412	1.00		927	25	1.00	
5–9	382	14	1.53 (0.73–3.18)	0.26	241	1.00 (0.81–1.22)	0.98	427	233	1.11 (0.90–1.36)	0.33	505	14	1.02 (0.52–2.00)	0.96
≥10	181	2	0.47 (0.11–2.10)	0.32	118	1.05 (0.81–1.37)	0.72	193	120	1.27 (0.97–1.65)	0.08	248	4	0.59 (0.20–1.75)	0.34
Total	1,274	34			822			1,434	765			1,680	43		

Bold type: 95% CI does not include 1.00. The ORs were adjusted for:

1. Age at diagnosis, region, and occupation.

2. Age at diagnosis, region, occupation, and parity.

* Carcinoma in situ.

** The number of cases for T2 (N = 1,047), T3 (176), and T4 (65).

## Discussion

In this large nation-wide follow-up study of 32,924 Swedish women with breast cancer, we found for postmenopausal cancer that multiparity was associated with a decreased risk of high-T class and distant metastases, while a late age at first or last childbirth increased the risk of high-T class. There was a general correlation between high-T class and distant metastases but not with lymph node metastases, which appeared not to be affected by reproductive factors. Risk of distant metastases was increased particularly in patients with a late age at first or last childbirth when the time interval between first and last childbirth was short, which is a typical reproductive pattern of educated women [33].

Our study used information on breast cancer patients diagnosed in the period 2002 to 2008, during which the histological classification system did not vary. Thanks to a complete cancer registration with verified histology, our study should be free of selection bias [28], [34]. Our findings on TNM classes, as prognostic data for breast cancer, suggested survival effects relating to reproductive factors but these could not be directly studied because the TNM classification was started first in 2002 [23]–[25]. Several limitations should be considered in interpreting our results. Some 24% of T, N and M classes were missing in the Cancer Registry. Neither were data available on age at menarche, breast feeding, obesity, oral contraceptive use, mammographic breast density, breast self-exam, hormone replacement therapy and hormone receptor status [10], [18], [19], [35].

Reproductive factors induce physiological changes in the mammary gland such as rapid proliferation of breast tissue [11]–[13], [36]. Previous studies reported that nulliparity and late age at first childbirth are related to aggressive tumor behavior [37]–[40]. Our results suggest that high-T class may mediate such effects: nulliparity was associated with the risk of high-T class, particularly in premenopausal ductal carcinomas and late age at first childbirth was associated with high-T class in postmenopausal ductal and lobular carcinomas. These findings suggest that physiological changes related to parity and age at first childbirth during pregnancy play a major role in the risk of high-T class and metastases in pre- and postmenopausal patients [9], [10]. Another explanation could be a non-attendance at invitational mammography screening, particularly among nulliparous women. Yet national mammography screening is attended by 81% of those invited in Sweden and mammography outside this program is also available thought the country [41], [42].

A study on 10,703 Danish women with breast cancer reported that early age at first childbirth was associated with a poor breast cancer prognosis [43]. We found accordingly that an early age at first childbirth increased the risk of high-T class in premenopausal ductal carcinoma (OR = 1.43) and increased the risk of metastases in postmenopausal cancer of particularly lobular carcinoma (2.23) while the effect on T class was opposite; this was the only instance where low T class was associated with the risk of metastases. According to Table 2, the discrepancy between low T class and metastasis was limited to those with early age at first childbirth who had a long time interval between first and last childbirth.

The association of age at last childbirth and breast cancer risk or prognosis is unclear [12], [13]. A case-control study reported an increased risk of breast cancer of 1.10 (95CI%: 1.03–1.16) for each 5-year increase in age at last childbirth [13]. We found that a late age at last childbirth increased the risk of high-T class in postmenopausal ductal and lobular carcinomas and premenopausal ductal carcinoma. One study reported that a late age at childbirth and nulliparity are more strongly associated with carcinoma in situ than invasive carcinoma in premenopausal patients [18]. In our data, the case numbers for premenopausal carcinoma in situ were small and only strong effects could have been detected. However, such strong effects (OR 8.00) were noted for postmenopausal lobular carcinoma in situ patients with a late age at last childbirth [44], [45].

A population-based study in Finland showed that short time interval between first and last childbirth is a protective factor for lobular carcinoma, but not for ducal carcinoma [14]. In our study, the only significant effect was on T class in postmenopausal ductal carcinoma. Pregnancy influences the level of estrogen and increases the risk of breast cancer in a short term, but decreases the risk in a long term [46]–[51]. Furthermore, pregnancy may also induce changes in hormone levels that may affect tumor progression in postmenopausal cases [52], [53]. Whether the level of estrogen influences the risk of high-T class and metastasis remains to be investigated.

## Conclusions

In summary, increasing parity was protective against high-T class and metastasis. Late age at first and last childbirth were risk factors for high-T class in almost all postmenopausal breast cancers and the effects were stronger for lobular than ductal histology. Low parity and long time interval between first and last childbirth were risk factors for distant metastases. The observed variation in the associations of reproductive factors with TNM classes suggests that hormonal and other physiological changes during pregnancy and menopause play an important role in determining T class and metastatic spread, with implications to prognosis. The current decline in parity and delayed age at first childbirth in many global populations may counterbalance the favorable achievements of prevention, screening and treatment of breast cancer.
